# Atypical Presentation of Wilson Disease: Unravelling a Clinical and Radiological Complexity in a Rare Case

**DOI:** 10.7759/cureus.54871

**Published:** 2024-02-25

**Authors:** Keta Vagha, Sham Lohiya, Jayant D Vagha, Priyanka Hampe, Ajinkya Wazurkar, Aashita Malik, Chaitanya Kumar Javvaji, Pankaj Banode

**Affiliations:** 1 Pediatrics, Jawaharlal Nehru Medical College, Datta Meghe Institute of Higher Education and Research, Wardha, IND; 2 Interventional Radiology, Jawaharlal Nehru Medical College, Datta Meghe Institute of Higher Education and Research, Wardha, IND

**Keywords:** d-penicillamine, trientine, copper metabolism, kayser-fleischer rings, hepatolenticular degeneration, wilson disease

## Abstract

Wilson disease (WD) is an autosomal recessive disorder marked by aberrations in copper metabolism, leading to its accumulation in vital organs such as the liver, brain, cornea, kidneys, and heart. While WD typically presents with hepatic symptoms in early childhood, neuropsychiatric manifestations are more prevalent during adolescence. This case report highlights an extraordinary instance of WD in an eight-year-old girl, characterized by intricate clinical and radiological features. The patient exhibited atypical symptoms, emphasizing the importance of recognizing diverse presentations of WD. Delayed diagnosis and treatment initiation can prove fatal in WD cases, underscoring the significance of awareness regarding these unusual clinical and radiological features to facilitate prompt intervention and prevent adverse outcomes.

## Introduction

Wilson disease (WD) is a hereditary condition that impacts the body's metabolism of copper. A mutation that causes hepatolenticular degeneration occurs in the ATP7B gene, which facilitates the excretion of copper into bile and supplies copper for the proper synthesis of ceruloplasmin. This blood protein transports copper [1]. The liver is impacted by WD since it is where dietary copper is metabolized. Additionally, excessive amounts of non-ceruloplasmin-bound copper build up in the brain, causing neuropsychiatric symptoms, and in red blood cells, causing hemolytic anaemia to appear, or in the cornea, causing the formation of Kayser-Fleischer (KF) rings. Still, it may also affect organs like kidneys, heart, and bones [2]. Hepatic symptoms typically manifest in early childhood, while neurological problems usually appear in adolescence [3]. Unrecognized impulsivity, behavioural problems, and subpar scholastic achievement are common neuropsychiatric signs; in children, neurological symptoms such as involuntary movements, speech disturbances, and autonomic dysfunction are seen [4]. Magnetic resonance imaging (MRI) is the most effective method for evaluating neuro-WD, which shows brain stem and basal ganglia grey matter typically involved due to copper build-up [5]. Here, we report a case of atypical Wilson disease in an eight-year-old girl who showed complexities in clinical presentation and radiological features exhibiting cortical involvement.

## Case presentation

**Figure 1:**
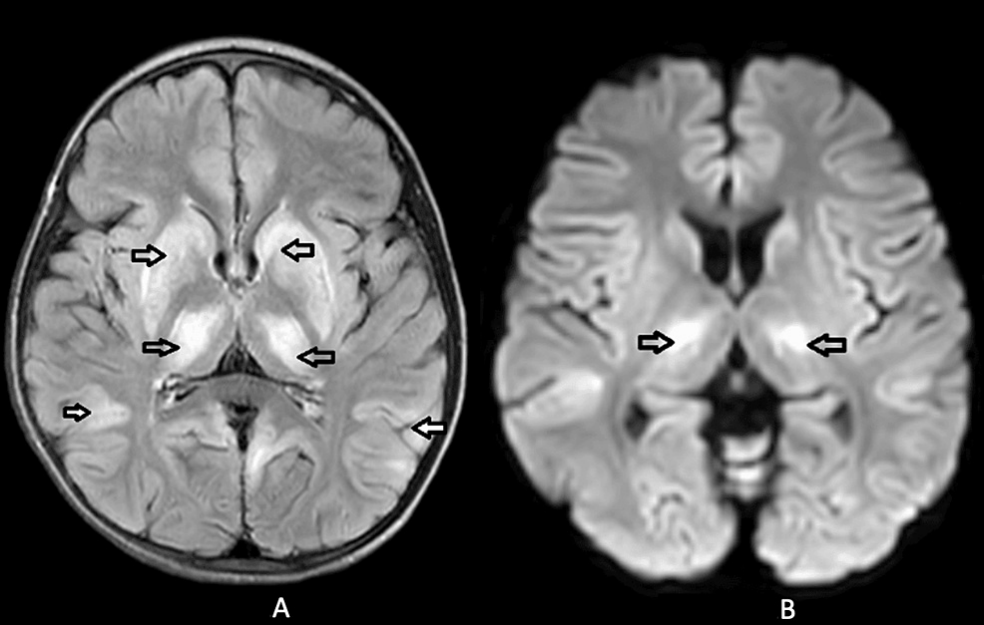
(A) T2 FLAIR hyperintensities (arrows) in the bilateral basal ganglia with cortical and subcortical hyperintensities in bilateral parietal lobes. (B) Diffused restriction in the bilateral basal ganglia suggestive of atypical Wilson disease (arrows) FLAIR, fluid-attenuated inversion recovery

An eight-year-old girl was brought to our tertiary care hospital in central India, presenting with a one-year history of difficulty in walking, tremors while grasping objects, a slow-paced gait, and frequent episodes of imbalance. A systemic examination revealed a wide-based, high-stepping gait, and swaying towards the left on Romberg's test. Normal tone and deep tendon reflexes were noted during the examination. Baseline blood investigations, including haemoglobin (12.1 gm%), total leucocyte count (7900/cumm), platelets (1.50 lacs/cumm), alanine transaminase (35 mg/dl), and aspartate transaminase (65 mg/dl) levels, were within normal limits. In light of the suspected Wilson disease, an ophthalmic assessment was conducted to identify KF rings, although their absence did not preclude the pursuit of further diagnostic measures. Given the heightened clinical suspicion, serum ceruloplasmin levels and 24-hour urinary copper excretion tests were subsequently ordered. MRI of the brain revealed non-enhancing altered signal intensity areas in bilateral cortical and subcortical regions, predominantly in bilateral fronto-parietal and temporal lobes, and symmetrical hyperintensities in bilateral basal ganglia, including corpus striatum, putamen, and thalamus (Figures 1A, 1B).

These neuroimaging findings indicated atypical Wilson disease, as it affected the subcortical region and involved the cortical region's grey matter. The patient was initiated on oral zinc supplements (75 mg/day), with plans to start D-penicillamine pending confirmation of elevated 24-hour urinary copper excretion and lower serum ceruloplasmin levels. After discharge, she returned a week later with complaints of vomiting, severe diffuse headache, persistent tremors, and difficulty in walking. An examination revealed slightly increased tone with exaggerated deep tendon reflexes. Serum ceruloplasmin levels were 8.5 mg/dl, falling below the typical 20-60 mg/dl range. This finding aligns with our clinical diagnosis of Wilson's disease. However, the 24-hour urinary copper excretion was measured at 24.3 mcg/24 hours, remaining within the normal range of 3-35 mcg/24 hours. This result deviated from the anticipated pattern, giving an atypical aspect to our diagnostic considerations.

The patient was started on a tablet of D-penicillamine (20 mg/kg/day) as Trientine was unavailable. Genome sequencing to detect ATP7B gene mutation was also sent because of the inconsistencies of other WD markers. Two weeks later, she presented with worsened headache, vomiting, severe neck pain, irritability, and echolalia. A lumbar puncture was performed to eliminate the possibility of meningitis, revealing normal levels of glucose, protein, and cellularity. Subsequent revaluation of markers for WD demonstrated an average serum ceruloplasmin level of 14.6 mg/dl while the 24-hour urinary copper excretion was at 6.90 mcg/24 hours, which persisted remarkably within the normal range for the second consecutive time, even following the administration of the D-penicillamine challenge test.

Cervical spine MRI was insignificant, and tab amitriptyline was started for the headache, showing gradual improvement. The patient was then shifted to tab Trientine (20 mg/kg/day), and D-penicillamine was stopped. Genome sequencing conclusively verified a heterozygous compound autosomal recessive inheritance pattern, confirming the diagnosis of atypical Wilson disease. The sequencing identified a variant in Intron 11, specifically c.2730+1G>A, affecting the 5' splice site. Additionally, within Exon 2, a variation denoted as c.728_732inv (p.Phe243_Asn244 delinsTyrTer) was observed. These genetic findings align with the established association of the presented case with Wilson's disease (OMIM#277900).

As symptoms improved, amitriptyline was stopped, zinc was reduced to 50 mg/day, and the patient was discharged on tab Trientine. Regular follow-ups revealed gradual improvement in neurological symptoms and tone. During one follow-up, the patient's elder brother, asymptomatic for hepatic or neurological issues, was screened. KF rings were absent, but the serum ceruloplasmin level was 10 mg/dl. He was started on D-penicillamine at 20 mg/kg/day and has shown no deterioration while on treatment. The patient remains compliant with regular follow-ups.

## Discussion

The presented case underscores the intricate nature of diagnosing and managing WD, mainly when it manifests with atypical neurological features in pediatric patients. Atypical Wilson disease poses a diagnostic challenge due to its rarity and the variability of clinical presentations. Our patient exhibited a unique combination of symptoms, including difficulty in walking, tremors, imbalance, severe headache and neck pain, and neurological deterioration, which prompted a comprehensive investigation and an interdisciplinary approach to treatment. Neuroimaging played a pivotal role in elucidating the complexities of the case. MRI results unveiled the involvement of not only the subcortical region, as typically observed in WD, but also the cortical region, thus signifying an atypical manifestation. The participation of both the subcortical and cortical areas, as observed in our patient, has been reported in a limited number of cases, highlighting the need for vigilance in recognizing such atypical presentations [6].

In our case, the absence of KF rings during the ophthalmic evaluation was initially perplexing. KF rings, though a hallmark of Wilson disease, may not be present in all cases, particularly in the early stages [7]. A similar absence of KF rings has been documented in pediatric cases with neurological presentations, reinforcing that their absence should not preclude the consideration of Wilson's disease [8]. Genetic confirmation through genome sequencing played a pivotal role in establishing the diagnosis. Heterozygous compound autosomal recessive inheritance was identified, consolidating the atypical nature of the case. A similar confirmation of atypical WD through genetic testing has been reported in the literature, highlighting the significance of molecular diagnostics in refining diagnostic accuracy [9].

Treatment strategies for WD primarily involve copper chelation and dietary modifications. Our patient initially responded positively to oral zinc supplementation, a standard therapeutic approach for the disease. However, the subsequent need for Trientine underscores the importance of individualized treatment regimens and ongoing monitoring [10]. Trientine, a copper-chelating agent, has demonstrated efficacy in neurological presentations of Wilson disease, aligning with its successful use in patients [11]. Our case report contributes to the growing body of evidence on the diversity of clinical and radiological presentations in Wilson disease, emphasizing the need for a nuanced approach to diagnosis and management.

This case represents the sole documented instance of WD characterized by consistently average values in 24-hour urinary copper excretion, an occurrence observed on two separate occasions. This distinctive presentation renders the case atypical. While the neurological variant of Wilson disease is rare in childhood, its recognition is vital for timely intervention and improved patient outcomes. Further research and the accumulation of similar case reports will enhance our understanding of the spectrum of WD presentations, guiding clinicians in providing optimal care for affected individuals.

## Conclusions

In conclusion, this case highlights the significance of identifying atypical neurological presentations of Wilson's disease in pediatric patients. Timely diagnosis, proper treatment, and continuous follow-up are imperative for effectively addressing the intricacies associated with atypical WD, thereby securing a favourable clinical outcome. The consideration of genetic mutations is warranted, particularly given the emphasized importance; however, the absence of Kayser-Fleischer rings suggests that the type of mutation may alter the presentation, necessitating further detailed examination.
